# A Case Report of General Anesthesia for Abdominal Surgery in Severe Cavitary Tuberculosis: Strategies to Minimize Barotrauma

**DOI:** 10.7759/cureus.103929

**Published:** 2026-02-19

**Authors:** David Meireles, Raquel Boto, Filipa Farias, Diogo Andrade

**Affiliations:** 1 Anesthesiology, Unidade Local de Saúde de São José, Lisbon, PRT

**Keywords:** anesthesiology, difficult airway intubation, mechanical ventilation, tuberculosis, videolaringoscopy with spontaneous intubation

## Abstract

A 37-year-old man with suspected tuberculosis and severe cavitary lung disease presented with an acute abdomen with pneumoperitoneum requiring emergent exploratory laparotomy. The patient presented with dysphagia, recurrent regurgitation, pancytopenia, treatment refractory elevated International Normalized Ratio (INR). His compromised respiratory status, likely to worsen under positive pressure ventilation, along with a high risk of aspiration of gastric content and coagulopathy during emergent abdominal surgery, required general anesthesia. In patients with extensive pulmonary tuberculosis and large lung cavernous lesions, general anesthesia increases the risks of dissemination of the disease, barotrauma, and impaired gas exchange, all of which may lead to respiratory failure. Available literature is scarce and focuses on employing regional anesthesia and avoiding mechanical ventilation whenever possible. Our plan included taking the patient to a negative pressure room with limited personnel wearing individual protective equipment; performing videolaryngoscopy with maintained adequate respiratory drive and endotracheal intubation under sedation and topical anesthesia of the vocal cords; careful titration of pressure support for normocapnia and normoxemia. Throughout surgery, peak pressures and respiratory mechanics were continuously monitored, and the patient maintained lung-protective tidal volumes and low airway pressures. He was successfully extubated in the operating room; no respiratory support was necessary in the postoperative period. By adapting available techniques to prioritize patient safety, general anesthesia with preserved spontaneous ventilation proved to be a viable and effective alternative when conventional approaches posed a significant risk. This strategy may be a safe option for similar patients undergoing open abdominal procedures.

## Introduction

Tuberculosis (TB) is an infectious disease caused by Mycobacterium tuberculosis and is primarily transmitted via airborne droplets generated by individuals with active pulmonary disease. In cases of active draining lesions, contact transmission is also possible. Although it predominantly involves the lungs, TB remains a major global health concern and continues to be the leading cause of death from a single infectious agent [1]. In 2023, 38,993 TB cases were reported across Europe. In the same year, 1458 new cases were documented in Portugal [2].

Patients with TB may present with a wide range of clinical manifestations. Pulmonary disease is the most common form and typically manifests as a chronic productive cough. Systemic constitutional symptoms - including fever, night sweats, and unintentional weight loss - are primarily mediated through the release of proinflammatory cytokines. Extrapulmonary TB can involve virtually any organ system; frequently affected sites include the lymphatic system (tuberculous lymphadenitis), the musculoskeletal system (bone and joint involvement), the abdomen (involving the gastrointestinal tract, peritoneum, lymph nodes, and visceral organs, either singly or in combination), and the central nervous system (tuberculous meningitis) [3].

Cavernous pulmonary lesions pose a particular challenge in the perioperative setting, especially among patients requiring emergency abdominal surgery. These individuals often have compromised lung architecture, impaired respiratory mechanics, and reduced pulmonary reserve, all of which increase susceptibility to postoperative pulmonary complications [4,5]. Thorough preoperative evaluation and tailored anesthetic planning are therefore essential. Regional anesthesia techniques - most notably epidural and spinal anesthesia - have been shown to reduce pulmonary complications in high-risk populations when compared to general anesthesia [6]. Case reports support the use of continuous epidural anesthesia combined with dexmedetomidine sedation for laparoscopic and a few laparotomy procedures in patients with suspected or confirmed TB, as these approaches provide effective analgesia while minimizing the risk of nosocomial transmission by avoiding airway manipulation and mechanical ventilation [7].

When general anesthesia cannot be avoided, particularly for upper abdominal surgery, the risk of postoperative pulmonary complications is substantially increased due to impaired mucociliary clearance, reduced lung volumes, and increased vulnerability to hypoventilation and infection. Additional patient-specific risk factors, including low body mass index (BMI), anemia, smoking history, and coinfections such as Aspergillus or nontuberculous mycobacteria, further contribute to postoperative morbidity [8]. Overall, patients with advanced pulmonary TB and cavernous disease who undergo abdominal surgery require meticulous preoperative optimization and the application of lung-protective perioperative strategies to mitigate the elevated risks of postoperative respiratory failure and mortality.

In patients with active pulmonary tuberculosis who present with additional risk factors - such as severe malnutrition with low BMI and coagulation disturbances - regional anesthesia may be contraindicated. In such cases, reliance on lung-protective ventilation strategies becomes essential to minimize postoperative respiratory complications and postoperative respiratory failure. In patients with heterogeneous lung compliance, such as severe cavitary tuberculosis, lung-protective ventilation strategies include the use of low tidal volumes, judicious application of positive end expiratory pressure (PEEP), avoidance of unnecessarily high inspired oxygen fractions, and restrictive use of recruitment maneuvers [9] to minimize the risk of volutrauma and barotrauma.

Ventilation strategies must be matched with an adequate airway management method. Patients with marked pulmonary architectural distortion, reduced respiratory reserve, and an increased risk of rapid desaturation during apnea present with difficult airway conditions. Futhermore in patients with active pulmonary tuberculosis, a high risk of aerosolization is anticipated. Awake fiberoptic intubation - or awake videolaryngoscopy in selected cases - using topical airway anesthesia provides a safer method of securing the airway while reducing hypoxemia, barotrauma, coughing, and droplet dispersion.

To limit the risk of opioid-induced respiratory depression in patients with diminished pulmonary reserve, multimodal analgesic strategies are essential. Superficial peripheral nerve blocks such as transversus abdominis plane (TAP) or rectus sheath blocks may still be considered, as they avoid the hemorrhagic risks associated with neuraxial or deep plexus techniques.

This case report outlines the anesthetic management of a patient with multiple large tuberculous cavernous lesions undergoing exploratory laparotomy, with emphasis on the strategies implemented to preserve respiratory function and ensure safe airway management.

## Case presentation

A 37-year-old male with no known prior medical history was brought to the emergency department of Hospital de São José (Lisbon) with altered mental status (Glasgow Coma Scale score of 8), irregular breathing, and severe hypoglycemia (blood glucose of 30 mg/dL). Following hypoglycemia treatment and after becoming more alert, he reported a nine-month history of progressive oropharyngeal dysphagia that substantially limited oral intake. Additional symptoms included profound fatigue, unintentional weight loss (current weight 30 kg), and night sweats requiring changes of clothing and bedding.

On examination, the patient was dyspneic but maintained a peripheral oxygen saturation greater than 94% on room air. He was tachycardic and normotensive. Pulmonary auscultation revealed globally reduced breath sounds. The abdomen was soft yet tender to palpation in the upper quadrants. According to hospital protocol, due to suspected *Mycobacterium tuberculosis* infection, he was admitted to a negative-pressure room, and all healthcare workers were provided adequate personal protective equipment.

Arterial blood gas analysis showed persistent hypoglycemia several hours after initial correction, hyponatremia (121 mmol/L), and hypocalcemia (0.89 mmol/L), without any acid-base disturbance. Laboratory evaluation showed anemia, leukopenia, an elevated International Normalized Ratio (INR), and prothrombin time without known anticoagulation. Liver enzymes, lactate dehydrogenase, and creatine kinase were mildly elevated. Inflammatory markers were significantly increased. Blood, sputum, and gastric juice samples were drawn for direct microscopy, cultures, and molecular assays, which later returned positive for *Mycobacterium tuberculosis*, confirming the diagnosis of bacilliferous tuberculosis. The human immunodeficiency virus screening test was negative. Results of the laboratory assessment are shown in Table 1. 

**Table 1:** Laboratory assessment on admission ALT  - alanine aminotransferase; aPTT - activated partial thromboplastin time; AST - aspartate aminotransferase; INR - International Normalized Ratio; LDH - lactate dehydrogenase

Parameters	Results	Reference values
Hemoglobin	9.6 g/dL	13.0 - 17.0 g/dL
Leucocites	3.44 x 10^9/L	4.5 - 11.0 x 10^9/L
Platelets	267 x 10^9/L	150 - 450 x 10^9/L
INR	2.39	0.8 - 1.2
Prothrombin time	28.4 seconds	9.4 - 12.5 seconds
aPTT	30.2 seconds	25.0 - 36.5 seconds
ALT	115 U/L	<40 U/L
AST	96 U/L	<41 U/L
LDH	800 U/L	135 - 225 U/L
Creatine kinase	610	<190 U/L
C-reactive protein	144 mg/L	<5.0 mg/L

**Figure 1:**
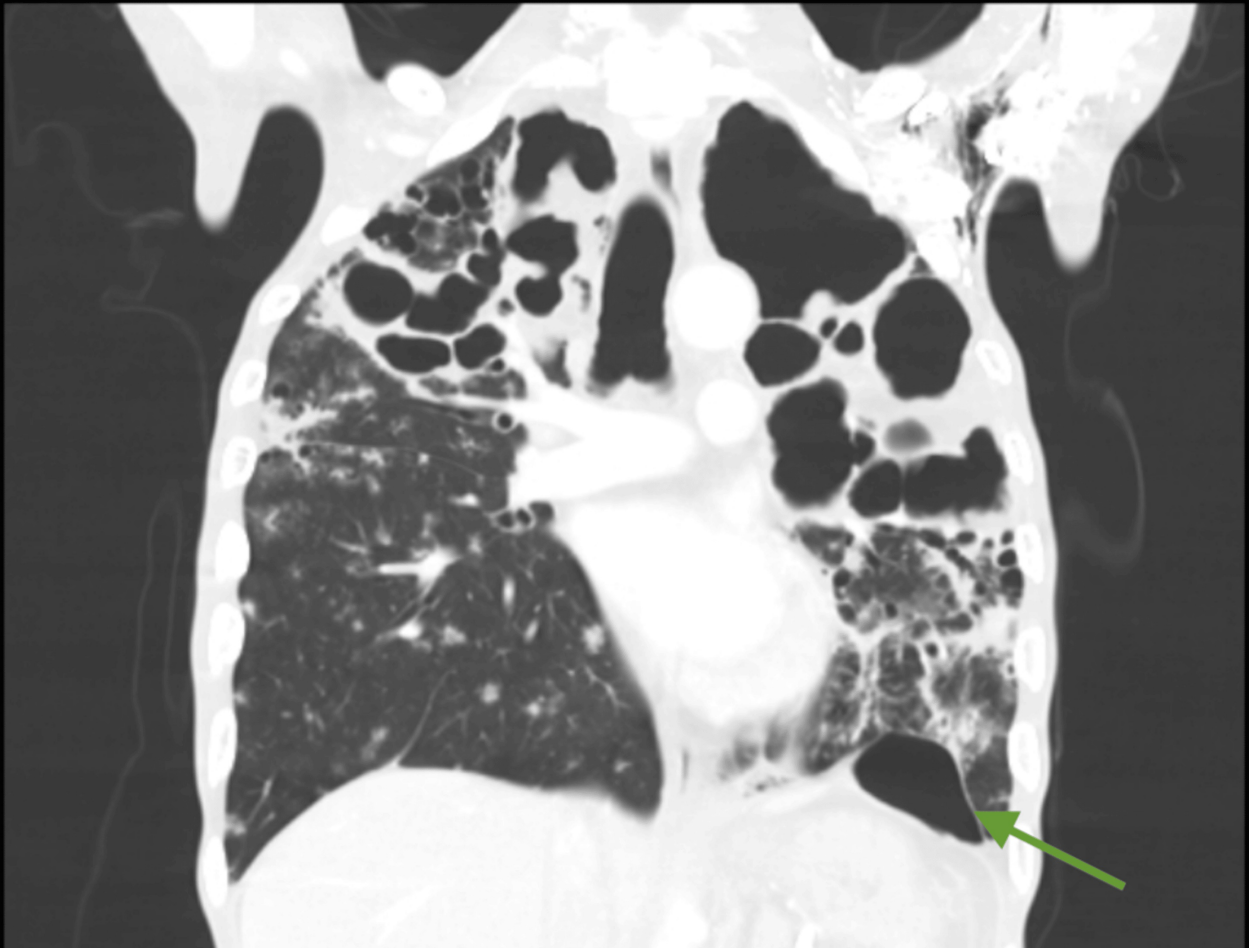
Coronal plane of the of chest on a contrast enhanced CT Extensive bilateral bronchopulmonary architectural distortion with multiple large, confluent cavitary lesions. The most affected regions included the right and left upper lobes, with the largest cavitation measuring approximately 8.5 × 7.7 cm. Green arrow: The left subpulmonic pneumothorax is visible between the heart and the diaphragm with the consolidation foci above it.

**Figure 2:**
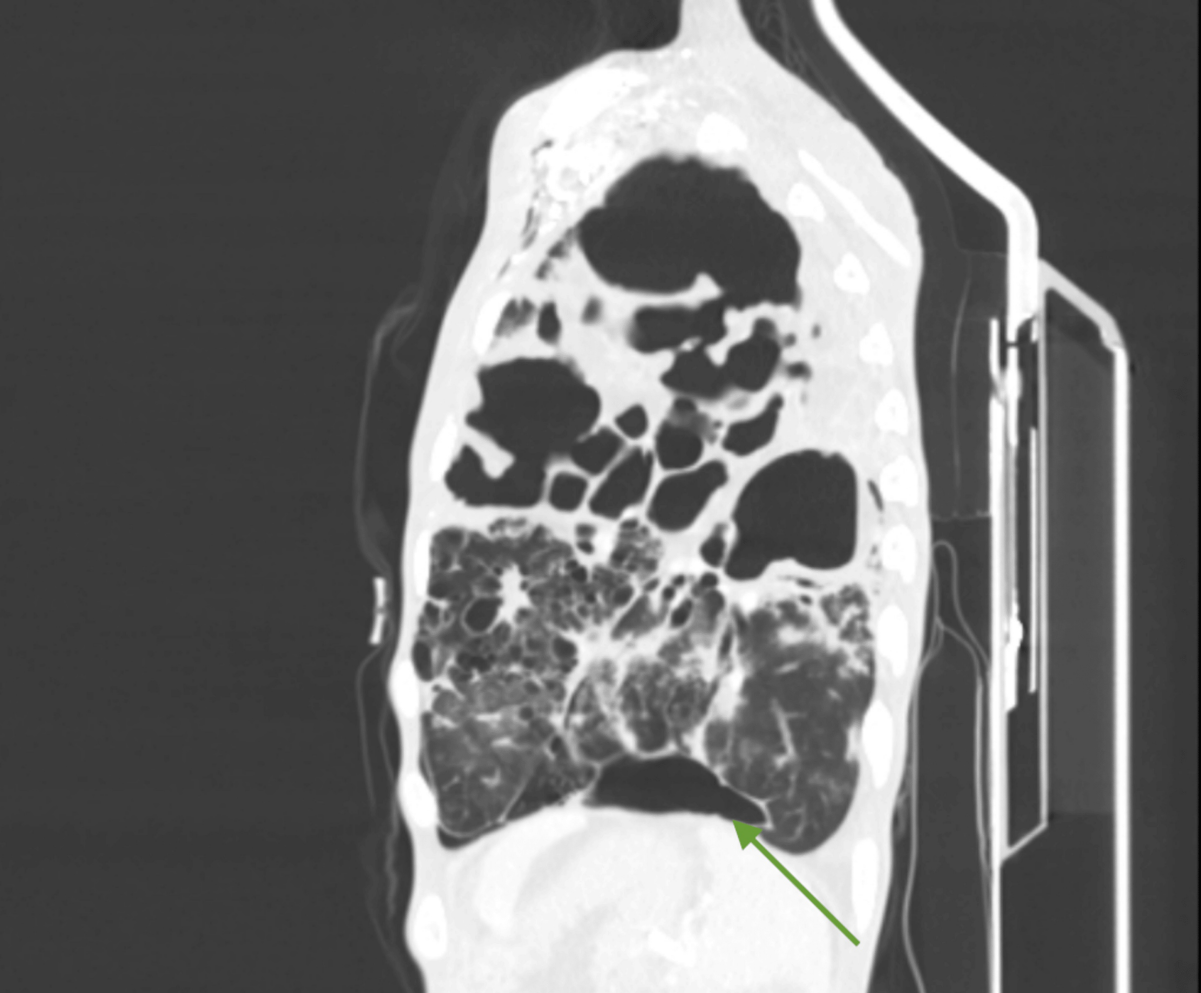
Sagital plane of the left hemitorax on the contrast enhanced CT Showing another perspective of the largest cavitations. Green arrow: the small pneumothorax between the diaphragm and the pericardium.

**Figure 3:**
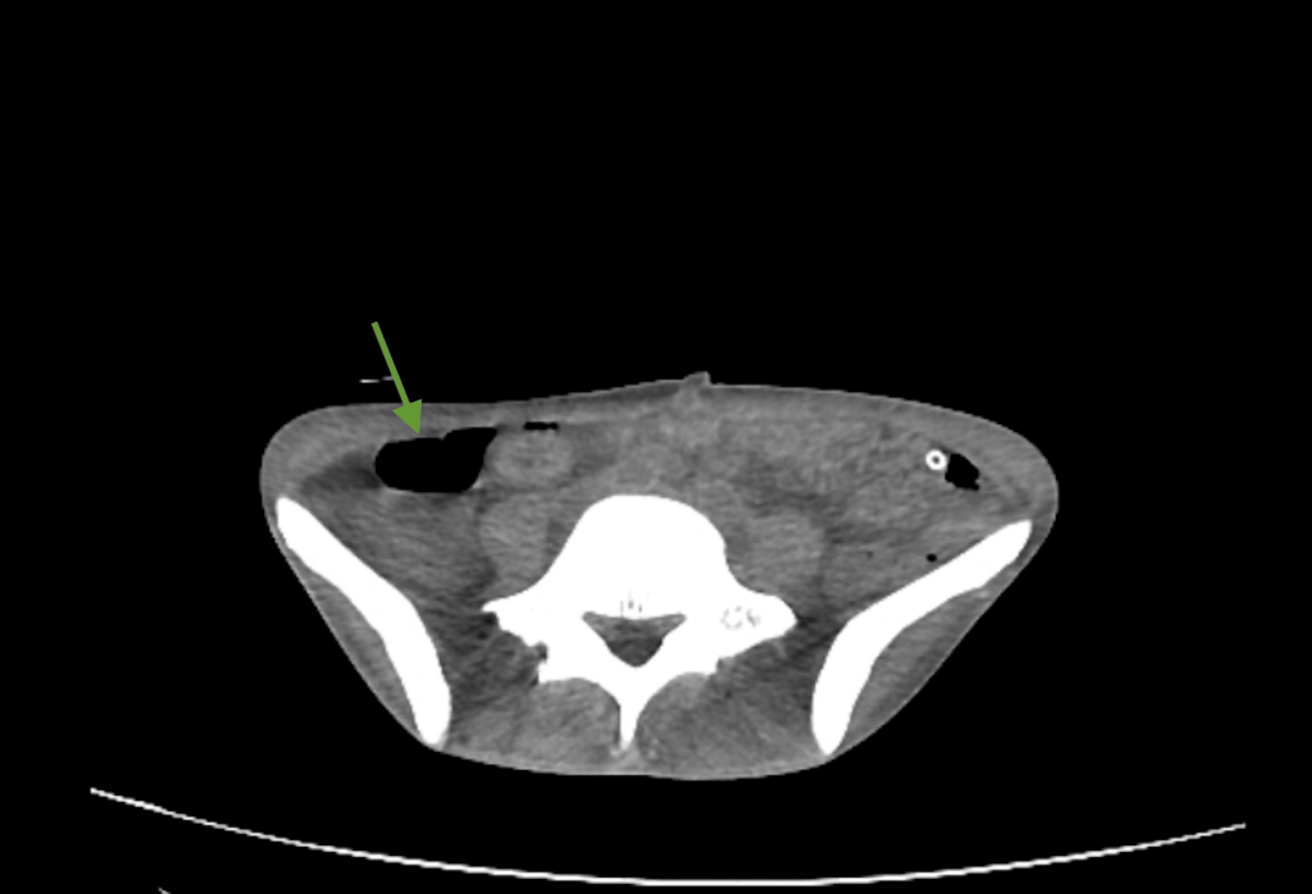
Transverse plane of the pelvis on a contrast enhanced CT showing the fluid levels of the ascites Green arrow: fluid levels of the ascites

**Figure 4:**
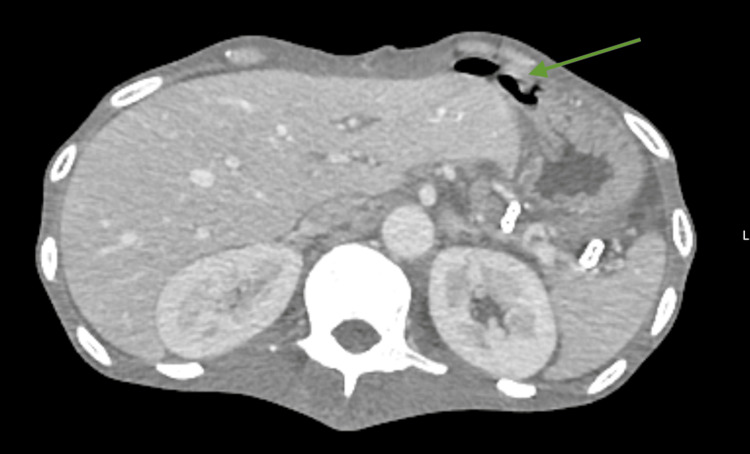
Transverse plane of the abdomen on a the contrast enhanced CT showing the pneumoperitoneum in the upper quadrants Green arrow: pneumoperitoneum in the upper quadrants of the abdomen.

Contrast-enhanced computed tomography (CT) of the chest and abdomen showed extensive bilateral bronchopulmonary architectural distortion with multiple large, confluent cavitary lesions. The most affected regions included the right and left upper lobes, with the largest cavitation measuring approximately 8.5 × 7.7 cm (Figures 1, 2). In the residual aerated parenchyma, there was diffuse impairment of ventilation with numerous coalescent centrilobular consolidative foci, accompanied by air bronchograms. These findings were highly suggestive of active bilateral infectious lung pathology, consistent with a mycobacterial etiology. A left paracardiac and left subpulmonic pneumothorax were also present, with a maximal thickness of 28 mm and containing internal septations. Abdominal and pelvic imaging revealed large-volume ascites and diffuse peritoneal enhancement compatible with active peritonitis (Figure 3). A moderate pneumoperitoneum was found, most prominent in the upper abdominal quadrants (Figure 4). Although the perforation site was not identified, the images strongly suggested gastrointestinal perforation, with a gastroduodenal ulcer being the most likely source. Assuming peritonitis due to perforation, the patient was admitted for an urgent exploratory laparotomy.

Preoperative optimization included the correction of the patient's persistent hypoglycemia and coagulation abnormalities. Despite administration of two units of fresh frozen plasma and intravenous vitamin K, the INR remained elevated (1.89). 

Considering the persistent coagulopathy, neuraxial anesthesia was deemed unsuitable and prompted induction of general anesthesia with airway protection while maintaining spontaneous ventilation. The patient was admitted to an operating room with negative pressure, and all healthcare personnel were required to wear disposable scrubs and N95 masks. Endotracheal intubation with spontaneous ventilation was performed via videolaryngoscopy under dexmedetomidine (50 µg IV over 10 min) and ketamine (40 mg IV) sedation. Topical anesthesia of the vocal cords was done using a mixture of 2.5 mL of 1% lidocaine and 2.5 mL of 0.5% ropivacaine. A HEPA filter was placed between the endotracheal tube and the breathing circuit, with an additional filter on the expiratory limb. Spontaneous breathing was preserved throughout the procedure. Pressure support ventilation was initiated, starting with inspiratory pressures of 10 cmH_2_O and a PEEP of 3 cmH_2_O, followed by cautious adjustment of airway pressures to maintain normoxia, normocapnia, and avoid exacerbation of preexisting air leak or barotrauma. Throughout the procedure, peak airway pressures were under 15 cmH_2_O, and driving pressures were between 10-12 cmH_2_O. General anesthesia was maintained with sevoflurane. Following endotracheal intubation and mechanical ventilation, an arterial line and central venous catheter were placed. Multimodal analgesia consisted of intravenous acetaminophen (1 g), metamizole (2 g), and ultrasound-guided bilateral transversus abdominis plane (TAP) block using 20 mL of 0.2% ropivacaine per side. Before the surgery started, there was a strategy briefing with the surgical team, and a compromise was reached on access to the surgical field.

Intraoperatively, the surgical team identified diffuse purulent peritonitis, along with a perforated gastric ulcer and two small lesions on the small intestine consistent with miliary implants. Biopsies from both the ulcer margin and mesenteric implants later tested positive for *Mycobacterium tuberculosis* complex (MTBC).

During surgery, the patient developed hemodynamic instability requiring vasopressor support with norepinephrine infusions up to 1 µg/kg/min. Although estimated intraoperative blood loss was approximately 50 mL, arterial blood gas sampling showed a decrease in hemoglobin to 7.9 g/dL, a hematocrit of 24%, and a lactate of 2.1 mmol/L. The hypotension appeared multifactorial, likely related to hypovolemia, progression of septic shock, and possible adrenal insufficiency. Given the concern for adrenal crisis, a fluid challenge and empirical hydrocortisone (100 mg IV) were administered, and one unit of packed red blood cells was transfused. Following these interventions, norepinephrine requirements decreased to 0.2 µg/kg/min. Throughout the procedure, the patient developed hypoglycemia (51 mg/dL), which was treated with IV 5% glucose-containing balanced crystalloid. The patient's net fluid balance was approximately +1450 mL despite an adequate urine output.

The patient was extubated in the operating room and transferred to a negative-pressure room in the intensive care unit (ICU) for continued monitoring with vasopressor support and no respiratory support. 

Antibiotics directed to MTBC (levofloxacin, rifampicin, and isoniazid) and empiric antibiotic therapy for the abdominal infection (amoxicillin and fluconazole) were initiated. Close monitoring of hemodynamics, electrolytes, and acid-base status was continued, and further endocrine evaluation was planned to assess adrenal function once clinically stable. During the 36-hour stay in the ICU, there was an overall improvement with the discontinuation of organ support, and acute phase laboratory parameters decreased. Given his clinical improvement, the patient was transferred to the Infectious Diseases Service, where the ongoing treatment was continued. A follow-up CT scan showed a small left hydropneumothorax with a few internal septations, smaller in size compared with the previous examination, with no clinical repercussions. No complications were registered in the postoperative period. 

## Discussion

Available literature on abdominal surgery for patients with severe pulmonary impairment [6] preferentially describes neuroaxial anesthesia along with sedation [7] to avoid airway manipulation and mechanical ventilation, as it is the anesthetic technique with a lower respiratory complication rate in selected high-risk patients [10-12]. However, the high risk of aspiration and coagulopathy contraindicated neuraxial anesthesia and prompted airway protection [13]. Therefore, general anesthesia with endotracheal intubation was presented as the safest option to safely undergo abdominal surgery. 

In this case, an endotracheal intubation with videolaryngoscopy and maintained respiratory drive while minimizing apnea and loss of ventilation is well supported in high-risk airway scenarios in community guidelines [14-16]. It preserved the patient's airway reflexes and respiratory drive [15] and reduced the risk of hypoxemia and the risk of aspiration of gastric content. 

The large cavernous lung lesions and pneumothorax, together with positive pressure ventilation, posed a high risk of respiratory complications. Since the patient maintained respiratory drive during the whole procedure, adequate sedation, ventilation trigger, and pressure support were cautiously titrated so as to align with lung-protective ventilation principles of tidal volumes between 6-8 mL/kg ideal body mass and mean airway pressures of less than 30 cmH_2_O. This strategy maintained an acceptable gas exchange and is recommended in the literature to reduce the risk of respiratory complications, especially pneumothorax, barotrauma, and atelectotrauma [16, 17]. 

Compared with prior reports emphasizing the preference of regional over general anesthesia, this case supports general anesthesia as a reasonable alternative when regional techniques are contraindicated, provided that spontaneous ventilation is preserved and ventilatory support is carefully titrated.

## Conclusions

Although the literature recommends neuroaxial anesthesia for abdominal surgery in patients with increased risks of aspiration and severe tuberculosis lung destruction, general anesthesia becomes necessary in selected cases, especially in cases where neuroaxial anesthesia is contraindicated, and the abdominal surgery eventually turns into an upper abdominal surgery and upper abdominal exploration which may require conversion to general anesthesia midway through the operation due to patient disconfort. This high-risk case shows that with an individualized strategy with preserved spontaneous breathing, cautious titration of pressure support, vigilant monitoring of airway pressure, appropriate sedation, and multimodal opioid sparing analgesia, these cases can safely undergo abdominal surgery under general anesthesia.
